# Evidence from clinical trials on high-risk medical devices in children: a scoping review

**DOI:** 10.1038/s41390-023-02819-4

**Published:** 2023-09-28

**Authors:** Kathrin Guerlich, Bernadeta Patro-Golab, Paulina Dworakowski, Alan G. Fraser, Michael Kammermeier, Tom Melvin, Berthold Koletzko

**Affiliations:** 1 LMU—Ludwig Maximilians Universität Munich, Division of Metabolic and Nutritional Medicine, Department of Pediatrics, Dr. von Hauner Children’s Hospital, LMU University Hospital, Munich, Germany; 2 Child Health Foundation - Stiftung Kindergesundheit, c/o Dr. von Hauner Children’s Hospital, Munich, Germany; 3 Medical University of Warsaw, Warsaw, Poland; 4 Department of Cardiology, University Hospital of Wales, Cardiff, Wales UK; 5 Department of Gerontology, School of Medicine, Trinity College Dublin, Dublin, Ireland; 6 European Academy of Paediatrics, Brussels, Belgium

## Abstract

**Background:**

Meeting increased regulatory requirements for clinical evaluation of medical devices marketed in Europe in accordance with the Medical Device Regulation (EU 2017/745) is challenging, particularly for high-risk devices used in children.

**Methods:**

Within the CORE-MD project, we performed a scoping review on evidence from clinical trials investigating high-risk paediatric medical devices used in paediatric cardiology, diabetology, orthopaedics and surgery, in patients aged 0–21 years. We searched Medline and Embase from 1st January 2017 to 9th November 2022.

**Results:**

From 1692 records screened, 99 trials were included. Most were multicentre studies performed in North America and Europe that mainly had evaluated medical devices from the specialty of diabetology. Most had enrolled adolescents and 39% of trials included both children and adults. Randomized controlled trials accounted for 38% of the sample. Other frequently used designs were before-after studies (21%) and crossover trials (20%). Included trials were mainly small, with a sample size <100 participants in 64% of the studies. Most frequently assessed outcomes were efficacy and effectiveness as well as safety.

**Conclusion:**

Within the assessed sample, clinical trials on high-risk medical devices in children were of various designs, often lacked a concurrent control group, and recruited few infants and young children.

**Impact:**

- In the assessed sample, clinical trials on high-risk medical devices in children were mainly small, with variable study designs (often without concurrent control), and they mostly enrolled adolescents.
- We provide a systematic summary of methodologies applied in clinical trials of medical devices in the paediatric population, reflecting obstacles in this research area that make it challenging to conduct adequately powered randomized controlled trials.
- In view of changing European regulations and related concerns about shortages of high-risk medical devices for children, our findings may assist competent authorities in setting realistic requirements for the evidence level to support device conformity certification.

## Introduction

Medical devices play a key role in the diagnosis and treatment of many diseases in children. The spectrum ranges from low-risk devices like dressing materials and wheelchairs to those of high-risk like catheters, ventilators, implants or pacemakers. While overall about 500,000 devices are available on the EU market,^1,2^ the number of those approved for the paediatric age group is not specified as there is no central database in Europe.^3^ Despite the advances in medical device technology, globally the market of medical devices is clearly dominated by devices for adults, while products specifically approved for paediatric use are in substantially lower number and often unavailable.^4^ Consequently, off-label use of adult versions of medical devices is often best practice, despite little to no evidence about suitability and safety of their use in children.^5,6^

In Europe, the regulation for approval of medical devices changed to improve the safety for patients by enhancing the regulatory requirements for evidence-based clinical evaluation of medical devices. Products marketed in the EU that were approved under the prior directives, as well as newly developed devices, will need to comply with the new Medical Device Regulation (MDR; EU 2017/745; https://eur-lex.europa.eu/eli/reg/2017/745/oj) by 26 May 2024, and fully so after the transition period which has been extended to December 2027 for high-risk medical devices.^7,8^

Meeting these new regulatory requirements can be challenging for manufacturers, especially with regard to clinical investigation of devices intended for patients in the paediatric age group. For many clinical conditions and diseases for which high-risk medical devices are intended, the numbers of patients are limited, events are rare, and the population under study is likely to be heterogeneous ranging from very small preterm infants to adolescents.^9^ In addition, both ethical considerations and parental concerns can make it complicated to recruit and enrol infants, children and adolescents, who constitute a vulnerable population, to trials.^10^ An additional barrier faced by manufacturers is high financial regulatory costs in Europe,^3,11^ with a low likelihood for achieving a return on investment due to the relatively small market for high-risk medical devices in the paediatric age group.

Together, these factors may result in market withdrawal of innovative medical devices for children, and lack of investment in further development and market introduction of new paediatric medical devices.^3^ While achieving and documenting the highest possible level of safety and efficacy for medical devices used in children is a laudable goal, at the same time providing access to innovative medical devices for the youngest patients and secure access to related state-of-the-art and potentially life-saving interventions remain equally important.

The project “Coordinating Research and Evidence for Medical Devices” (CORE–MD; https://www.core-md.eu/) is an EU Horizon 2020 project that reviews methodologies for the clinical investigation of high-risk medical devices, including those applied specifically in children, in order to recommend an appropriate balance between efficacy, safety and innovation.^11^ As part of this project, we systematically summarize published clinical evidence on high-risk medical devices, namely available evidence from clinical trials in children, in order to identify and describe methodologies applied in this research area. Given our broad review questions and thus the exploratory character of the review, we conducted a scoping review. This approach is recommended when the purpose of the review is to *“scope a body of literature, clarify concepts or to investigate research conduct”*,^12^ and it is typically used to provide an overview and map of the available evidence in a given field and to identify knowledge gaps.^12^

## Methods

This scoping review was performed in accordance with the previously developed protocol, which was registered and published at the Open Science Framework (https://osf.io/uzekt).^13^

We conducted this review in accordance with the methodology of the Joanna Briggs Institute’s (JBI) Reviewers’ Manual^14^ and report the results following the PRISMA-ScR guidelines^15^ (Preferred Reporting Items for Systematic reviews and Meta-Analyses extension for Scoping Reviews).

### Inclusion and exclusion criteria

#### Participants

The study population of interest was children and young people from different age-groups covering the range from 0 to <21 years, including preterms, neonates, infants, toddlers, children and adolescents, with any medical condition as an indication for the use of a specific medical device. Mixed population studies that involved both children and adults were also eligible for inclusion.

#### Concept

Medical devices, including paediatric medical devices, are categorized in different risk classes according to the EU Medical Device Regulation (MDR), as well as to the U.S. Food and Drug Administration (FDA) regulations. However, the classification rules are different and some products may fall into different risk categories. The focus of this review was on high-risk medical devices. According to the MDR, high-risk medical devices are “class III implantable devices and class IIb active devices that are intended to administer or remove medicinal products from the body”.^16^ According to the FDA, high-risk medical devices are class III devices that “usually sustain or support life, are implanted, or present potential unreasonable risk of illness or injury”.^17^ Examples of high-risk medical devices are prosthetic heart valves, closed-loop insulin delivery systems, defibrillators or deep-brain stimulation. In our review, studies on class IIb and III medical devices according to the MDR, and on class III medical devices according to the FDA were considered for inclusion.

For feasibility reasons, we focused on selected medical devices, based on a pre-defined list of high-risk paediatric medical devices from cardiology, diabetology, orthopaedics and surgery. This selection is in line with the similar reviews done by the CORE-MD consortium for adult populations^18–20^ and it covers clinical specialties (cardiology; clinical chemistry that includes insulin pumps and glucose sensors) that are frequently represented among approved devices in children.^21^ In Europe, medical devices are not listed centrally. The European Database on Medical Devices (EUDAMED) will be mandatory in the future to track all devices placed on the EU market under the MDR, but is still under development. Therefore, we developed the list of devices of interest (Supplementary Table 1) using sources based on FDA records.

We used the device list provided by ref. ^21^, who identified all high-risk medical devices with paediatric age indications listed in the FDA Premarket Approval (PMA) database from inception to February 2020 in their study. Additionally, we supplemented this list by searching the following FDA resources:

- Premarket Approvals (PMA) database: https://www.accessdata.fda.gov/scripts/cdrh/cfdocs/cfpma/pmasimplesearch.cfm (from March 2020 to June 2022)
- Annual Reports to Congress on Premarket Approval of Paediatric Uses of Devices, covering approved PMA and Humanitarian Device Exemption (HDE) applications (available from 2008 to 2017)
- Humanitarian Device Exemption (HDE) database (from 2018 to June 2022)

In this scoping review, we investigated designs and methods applied in clinical trials with the use of a high-risk medical device in children as an intervention. According to the International Committee of Medical Journal Editors (ICMJE) a clinical trial is “*any research project that prospectively assigns people or a group of people to an intervention, with or without concurrent comparison or control groups, to study the relationship between a health-related intervention and a health outcome*”.^22^ While there are many other definitions of a “clinical trial”,^23,24^ in any case a clinical trial is an interventional study and thus differentiates itself from studies of observational design.

Due to the nature of this review, the list of outcomes of interest remained open but included the following:

- Country; single- or multicentre, national or international study
- Study design (e.g., controlled clinical trial, crossover trial, single-arm interventional study)
- Sample size and proportion of paediatric participants
- Target population characteristics (age, sex)
- Type of device and indication for its use
- Assessed study outcomes (e.g., safety, performance, efficacy, patient reported outcomes)
- Approving body
- Funding (e.g., industry sponsorship)

#### Context

We included any clinical trials’ reports on high-risk medical devices in children, including those on pre- and post-market clinical investigation. No restrictions were applied in terms of study setting or device indications for use, with areas of application including cardiology, diabetology, orthopaedics and surgery.

#### Types of sources

Clinical trials of any design (e.g., randomized and non-randomized controlled clinical trials, interventional studies without concurrent controls, before–after studies, crossover trials) and qualitative studies focused on the intervention being trialled, were eligible for inclusion. Evidence from systematic reviews and observational studies (prospective, retrospective) was not of interest. Conference abstracts, commentaries, editorials, letters and book chapters were excluded.

### Search strategy

We searched the following electronic medical databases: MEDLINE (PubMed) and EMBASE (Ovid). Database-specific search strategies were developed based on the predefined list of high-risk medical devices, with the use of trade and generic devices’ names and clinical trials search filters. The timeframe for our search was from 1st January 2017 to 9th November 2022. We restricted our search to sources and papers published in English language only. The detailed search strategy is provided in Supplementary Table 2.

### Literature selection

Records identified after applying our search strategy were uploaded into reference manager EndNote (Version X8 and 20) and duplicates were removed. Titles and abstracts were screened against the inclusion criteria by one reviewer (PD, KG, MK).^25^ This process was pilot-tested on a selected subgroup of references with the involvement of all reviewers. Full text articles were obtained for abstracts that needed to be included or that appeared unclear. They were independently evaluated by two reviewers (PD, KG, MK). Any disagreements or uncertainties regarding inclusion were resolved through discussion and record assessment by the third independent reviewer (BPG).

### Data extraction

Data extraction was performed manually by two independent reviewers for each included article using a pre-specified data extraction form, and it was later cross-checked for any discrepancies. We extracted information on authors, year of publication, study setting and design, sample size, participant demographic characteristics (age, sex), study aim, medical device characteristics (trade and generic name, medical condition the device is intended for), assessed study outcomes, funding, if the device is on the market and if the study serves for clinical evaluation purposes.

### Data analysis

We used basic descriptive statistics (e.g., frequencies, proportions) to summarize the study designs, sample sizes and proportion of paediatric participants within the study sample, the study setting and population characteristics (with main emphasis on the age groups), the type of devices and their distribution across studied clinical specialties, assessed study outcomes and sources of funding.

## Results

**Fig. 1:**
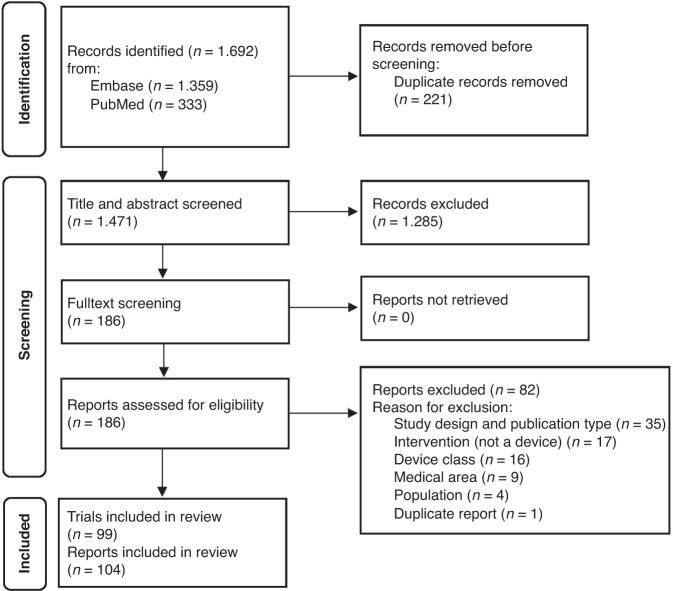
Flow diagram of the scoping review stages. *From:* Page MJ, McKenzie JE, Bossuyt PM, Boutron I, Hoffmann TC, Mulrow CD, et al. The PRISMA 2020 statement: an updated guideline for reporting systematic reviews. BMJ 2021;372:n71. doi: 10.1136/bmj.n71.

Of 1692 records identified, 104 reports^26–129^ on 99 trials were included in this scoping review (Fig. 1). We considered multiple reports as one trial if the population enrolled was fully the same. Excluded studies together with reasons for exclusion are presented in Supplementary Table 3 and details on characteristics of each included study in Supplementary Tables 4 and 5.

### Study setting

**Fig. 2:**
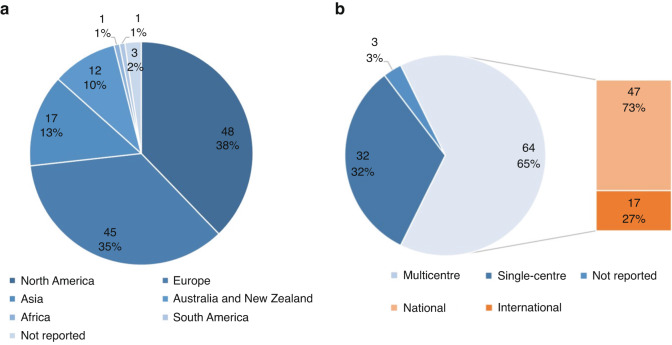
Trials distribution by continent and centre. **a** Trials distribution by continent* **b** Trials distribution by centre. *16 trials were multi-country studies. Therefore, the sum of trials is not 99 in Fig. 2a.

90% of the included studies were conducted in countries of very high Human Development Index (HDI), mainly in North America (38%) and Europe (35%). 65% of the studies were multicentre. Of those, 73% trials were conducted within one country and 27% enrolled participants from different countries. Distributions of the trials across continents and by centre are shown in Fig. 2a, b.

### Evaluated medical devices

Most of the included trials (*n* = 87, 88%) evaluated the use of medical devices from the clinical specialty of diabetology, followed by cardiology (*n* = 12, 12%). These included closed loop systems, glucose monitoring devices and insulin pumps. We identified no trials that had evaluated the use of medical devices from the clinical specialities of paediatric orthopaedics or paediatric surgery. The list of evaluated medical devices (generic names) by clinical specialty is provided in Table 1. Eight trials did not report the trade name of the evaluated device or the exact model that had been evaluated. 53% of the trials studied a medical device already available on the market. The other trials studied a medical device that was not on the market, or else information about the status of the device was unclear.

**Table 1:** Medical devices assessed in the included studies.

Clinical specialty	Medical device	*N*	%
Diabetology	Closed loop insulin delivery system	24	24
	(Advanced) hybrid closed loop insulin delivery system	22	22
	Open loop control system	1	1
	Predictive low-glucose management (PLGM) system	4	4
	Continuous glucose monitoring (CGM)	27	27
	Continuous subcutaneous infusion of insulin (CSII), insulin pump	9	8
Cardiology	Atrial septal defect occluder	4	4
	Transcatheter pulmonary valve	3	3
	Transcatheter heart valve	1	1
	Ablation catheter with mini-electrodes	1	1
	Covered stent	1	1
	Fully bioabsorbable pulmonary valved conduit	1	1
	Novel expanded polytetrafluoroethylene-based valved conduit	1	1

### Population

60 trials (61%) enrolled only paediatric populations (participants <21 years of age). The remaining 39 trials (39%) evaluated the device of interest in a mixed population, consisting of both children and adult study participants. Within the studies with mixed populations, 64% (*n* = 25) reported the exact number of children with a proportion from 10% to 89% (median 52%, interquartile range, IQR 45–65%).

We categorized the age groups of interest as followed: neonates from birth through the first 28 days, infants from 29 days to 2 years of age, children from 2 years to 12 years of age, and adolescents from 12 to 21 years, according to ref. ^21^ and the FDA classification.^130^

**Fig. 3:**
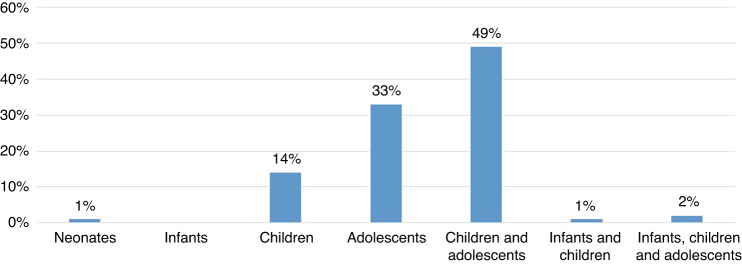
Distribution of age groups. Distribution of age groups of patients examined in the included studies.

Most studies included children and adolescents (49%), followed by adolescents (33%) or children only (14%) (Fig. 3). Of the remaining reports, two studies (2%) enrolled participants of a broader age range including infants, children and adolescents, one report (1%) included infants and children, and one report (1%) enrolled neonates.

Overall, 51 studies (51.5%) included multiple paediatric age groups, and 48 studies only a single paediatric age group (48.5%). Among the studies with mixed populations (*n* = 39), 36 studies (92%) included adolescents over 18 years of age.

### Study designs

The largest single category of included studies were randomized controlled trials (RCTs) (38%), followed by baseline-controlled trials (before-after studies) (21%) and trials of crossover design (20%) (Table 2). Of all controlled clinical trials and crossover trials, 90% were randomized. All crossover trials and most of the RCTs were open-label studies, with blinding (single or double) applied in only 13% of RCTs.

**Table 2:** Types of study designs in the included studies.

Study design	*N*	%
Randomized controlled trials, RCTs	38	38
Nonrandomized controlled clinical trials	4	4
Crossover trials	20	20
Before–after studies/baseline-controlled trials	22	21
Clinical performance studies with reference device	7	7
Uncontrolled trials	4	4
Cluster randomized controlled trials	1	1
Interventional studies with historical controls	1	1
Qualitative studies on intervention being trialled	2	2

### Sample size

**Fig. 4:**
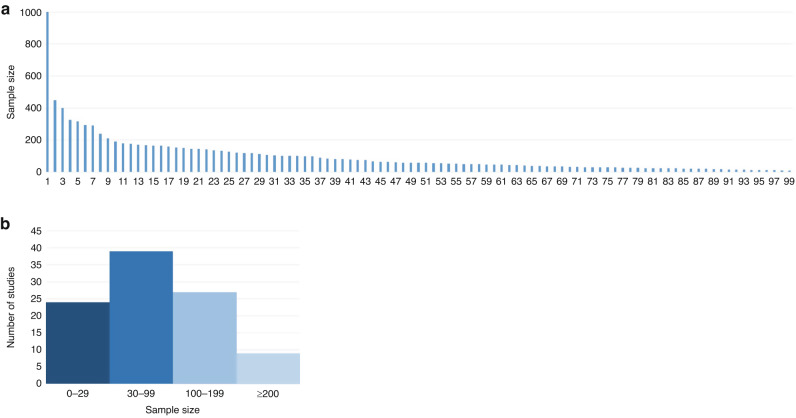
Sample size distribution. **a** continuously (**b**) per category.

The sample size varied across the studies and ranged from 10 to 1000 participants. Most of the included trials were small, with the median number of participants 59 (IQR 30–124.5) and with a sample size <100 participants in 64% of the studies. Figure 4a, b shows the distribution of the sample sizes of the included studies (continuously, per category: 0–29, 30–99, 100–199, ≥200).

In the subgroup of studies (*n* = 60) that enrolled only a paediatric population (participants <21 years of age), the median sample size was 48 (IQR 24–102). The median number of study participants was similar in the paediatric studies from the field of diabetology only (57 trials, median sample size of 50, IQR 24–105), but was significantly lower in studies from the field of cardiology (3 trials, median sample size 17, IQR 14.5–30.5).

### Assessed outcomes

Most studies assessed the efficacy or effectiveness of the device used (79%), and the safety of the intervention (73%). Patient-reported outcomes were assessed in 24% of the trials. 23% of trials focused on the performance of the device. Usability was examined only in studies from the field of diabetology. Table 3 shows all different types of outcomes assessed in the included studies.

**Table 3:** Types of the study outcomes assessed in the included trials.

Types of the study outcomes assessed	*N*	%
Efficacy/Effectiveness	78	79
Safety/Adverse events	72	73
Patient reported outcomes, PROM	24	24
Performance/Accuracy	23	23
Usability/User experience	19	19
Feasibility	6	6
Cost evaluation/cost-effectiveness	2	2
Interoperability	1	1
Other	6	6

### Funding

32 trials (32%) were industry funded, and 42 trials (43%) were partly industry funded meaning that devices were provided by industry for free or at a discounted price. Of all studies in which industry was involved in the funding, 10 trials specifically mentioned that they were investigator-initiated. 13 trials (13%) were non-industry funded. No funding had been received, or funding was not reported in 12 trials (12%).

## Discussion

### Summary of findings

This study provides an overview on designs and methodologies applied in a systematically selected sample of recent clinical trials evaluating high-risk medical devices in infants, children and adolescents. Our sample was dominated by devices from the clinical specialty of diabetology, while we identified only few studies of cardiology devices and none of orthopaedic or surgery devices in children. Of all identified clinical trials, 38% were RCTs. Remaining trials were of various study designs, often without a concurrent control group, and included crossover trials and before-after studies. Other study characteristics such as small sample sizes and multicentricity were common. Identified studies were mostly conducted in adolescents and older children, with a very low number in neonates, infants and young children.

We based our search on devices with an approved indication from the FDA for use in paediatric patients, although mostly, they had not been approved for the youngest children.^21^ This may to some extent explain the fact that we found nearly no studies performed in infants and young children. Overall, both low number of devices approved for this young age group and low number of clinical trials identified by us that targeted this population may primarily indicate greater barriers in obtaining clinical evidence in this group. Both ethical aspects and parental concerns can hamper participant recruitment and enrolment and make it more difficult to perform clinical studies in children, particularly in younger age groups, also because of limited number of patients available and rarity of events.^9,10^ These barriers likely influence not only the number of studies, but also their design.

Only 38% of the clinical trials within our sample were RCTs, which is similar to the recent report on clinical evidence for FDA first-time approved high-risk therapeutic devices, showing that trials with randomization accounted for 49% of pivotal studies for paediatric devices.^131^ Unsurprisingly, nearly all of the identified RCTs had been conducted among patients with type 1 diabetes, one of the most common chronic diseases in children.^132^ It is also not unexpected that all crossover trials in our sample were from the field of diabetology, as this study design provides a high level of evidence in patients with chronic diseases, such as diabetes, with a temporary/reversible effect induced by a device (e.g., glycemic control), and at the same time allows for significant sample size reduction because study participants serve as their own controls.^133^ In contrast, within our small sample of studies from the specialty of cardiology, the leading study designs were uncontrolled studies and before-after studies. Considering the type of indications for high-risk medical device use in paediatric cardiology, such as rare congenital heart defects often requiring urgent and life-saving interventions at a very young age, these findings appear understandable. Other trial design features, such as mixed population under study, multicentricity or small sample sizes that characterized trials within our sample are also likely to be derived from the above-mentioned barriers in study participant recruitment.

We identified a substantially higher proportion of studies conducted in type 1 diabetes patients, than in the other specialities that we included, which again is likely to be at least partly explained by the relatively high prevalence of this disease. Moreover, evaluated devices from this clinical field were basically identical to those used in adults. For manufacturers, it seems to be financially more attractive to conduct studies on devices with large sales volumes, and with long-term use of the devices, which can provide a significant profit margin. We speculate that this is an additional reason why most included studies were performed on commercially attractive devices such as diabetic devices. Additionally, diabetic devices tend to be subject to health technology assessments for reimbursement purposes and, therefore, need more studies of high quality confirming their effectiveness to meet the reimbursement standards set by national authorities. Interestingly, we did not identify any studies of orthopaedic devices in children. However, orthopaedics, with a low number of devices indicated for children within this subspecialty, is not one of the leading fields for devices in paediatrics compared to cardiology, clinical chemistry, ophthalmology or otolaryngology^21^ and in contrast to devices in adults.

As anticipated, most often reported study outcomes in our sample were efficacy or effectiveness of the device used, and safety of the intervention. Nearly one quarter of studies assessed patient-reported outcomes, which provide valuable information about the impact of a treatment from the perspective of a patient that often cannot be captured by clinical measures.^134^ Our results indicate that there is room left for improvement and inclusion of patient-reported outcomes. As the MDR also mentions *“meaningful, measurable patient-relevant clinical outcome(s),*^6^ under clinical benefit to be assessed, this can increase the inclusion of these types of outcomes in future studies.

Finally, our findings show a substantial contribution of commercial manufacturers in the identified clinical trials, which comes with clear benefits but also with concerns about potential bias introduced in company-sponsored studies. A physician-initiated industry-sponsored study model is among the solutions to consider in order to reduce bias associated with medical device companies involvement into device research.^135^ Further, ensuring good clinical practice by applying the ISO 14155 standard and requiring sponsors to publish all clinical investigation reports as newly required by the MDR, are means of defense against possible bias.

Given the concerns about limited availability of some high-risk paediatric medical devices in Europe,^3,136^ European clinical experts have recently developed recommendations on clinical investigation and evaluation of high-risk medical devices for children.^137^ Findings obtained from this review assisted in the development of these recommendations.

### Strengths and limitations

To our knowledge this is the first systematic summary of the methodologies applied in clinical trials on high-risk medical devices in children, not limited to studies identified through FDA sources and exclusively supporting FDA approval of medical devices, but exploring published evidence from various settings and regulatory systems. In addition, a wide range of devices from different clinical specialties was covered in our search. We applied rigorous methods for the review conduct, as proposed in JBI Reviewers’ Manual, with respect to different review stages, including study identification and selection process, data extraction and synthesis.

Although due to feasibility reasons we did not apply free text words and standardized keywords to all concepts covered in our search strategy, both generic and trades names of devices of interest were covered in attempt to identify relevant trials. While we applied validated search filters for clinical trials, we acknowledge that their sensitivity to identify non-randomized trials may be lower as compared to RCTs.^138^ Therefore, we cannot exclude that some potentially eligible studies might have been omitted by us. Finally, our review was limited to studies published in English in the last 5 years, and did not include unpublished or grey literature or potentially existing confidential data used for device evaluation purposes. However, it should be noted that the aim of this scoping review was to obtain a representative sample of recent clinical trials on high-risk medical devices in children, rather than to identify the totality of evidence. Our selection of devices of interest was based on the U.S. FDA sources as there is no central European database of (paediatric) medical devices,^3^ which can be considered as a limitation of this review. Other challenges in the review conduct concerned determination of device class, complicated by the different device classification systems (e.g., FDA vs. EU criteria for high-risk medical devices), changes in classification over time, or no central source of information regarding device class in Europe.

## Conclusion

While RCTs are considered the gold standard for the effectiveness and safety assessment of a medical intervention, they may not always be feasible in clinical investigation of medical devices in children. Clinical trials of other designs, as those identified in our review, offer a compromise between the highest level of evidence and the lack of evidence. Paediatric devices require specific considerations and have unique barriers to their development. The findings from this scoping review may assist regulators and competent authorities in setting achievable and context-tailored requirements for clinical evidence supporting device conformity. Urgent actions are needed in Europe to ensure both the safety and the continued availability of devices that are essential to treat sick children. Capitalizing on respective evidence-based summaries supports regulatory decision-making processes.

### Supplementary information

PRISMA-ScR-Checklist

Supplementary Material_Table 1+2+4+5

Supplementary_Material_Table_3

## Data Availability

In this study, we used only publically available, published data.
