# Supplementary material for: Evidence from clinical trials on high-risk medical devices in children: a scoping review
Source: Pediatr Res. 2023 Sep 28;95(3):615–24. doi: 10.1038/s41390-023-02819-4 (PMC10899114; doi:10.1038/s41390-023-02819-4)
Supplement: Supplementary file 3 — Supplementary_Material_Table_3 [file 41390_2023_2819_MOESM3_ESM.pdf]

Supplementary Material

Evidence from clinical trials on high-risk medical devices in children: a scoping review

Kathrin Guerlich[†], Bernadeta Patro-Golab[†], Paulina Dworakowski, Alan G. Fraser, Michael Kammermeier, Tom Melvin, Berthold Koletzko[*]

[†]**Equal contribution and first authorship:** These authors contributed equally to this work and share first authorship.

***Corresponding Author:**
Berthold Koletzko, MD, PhD, Division of Metabolic and Nutritional Medicine, Department of Pediatrics, Dr. von Hauner Children's Hospital, University Hospital, LMU Munich, Lindwurmstr. 4, D-80337 München. Email: berthold.koletzko@med.uni-muenchen.de. Phone: +49 (0)89 4400 53967. Fax: +49 (0)89 4400 57742.

Supplementary Table 3: Full-text publications excluded with reason

[References]	Reason for exclusion
[1-35]	Study design and publication type
[36-52]	Intervention (not a device)
[53-68]	Device class
[69-77]	Medical area
[78-81]	Population
[82]	Duplicate report

References

1 Alawadi, F. et al. Impact of Provision of Optimum Diabetes Care on the Safety of Fasting in Ramadan in Adult and Adolescent Patients with Type 1 Diabetes Mellitus. *Diabetes Research and Clinical Practice* **169 (no pagination)** (2020).

2 Bozat, A. D., Akgul, S., Ertugrul, I. & Karagoz, T. Impact of Pacemakers and Implantable Cardioverter Defibrillators on the Psychosocial Functioning of Paediatric Patients. *Cardiology in the Young* (2020).

3 Clin, J. et al. Biomechanical Comparison of the Load-Sharing Capacity of High and Low Implant Density Constructs with Three Types of Pedicle Screws for the Instrumentation of Adolescent Idiopathic Scoliosis. *Spine Deformity* **7(1)**, 2-10 (2019).

4 Deng, Q. et al. Comparison of the Efficacy of Different Insulin Administration and Blood Glucose Monitoring Methods in the Treatment of Type 1 Diabetes Mellitus in Children. *Evidence-based Complementary and Alternative Medicine* **2022 (no pagination)** (2022).

5 El Samahy, M. H., Salah, N. Y., Abdeen, M. S. & Falastin, B. R. K. Psychosocial Aspects of Continuous Subcutaneous Insulin Infusion in Children with Type 1 Diabetes in Egypt; a Limited Resources Country Perspective. *Diabetology and Metabolic Syndrome* **14(1) (no pagination)** (2022).

6 Elbalshy, M. et al. The Miaomiao Study: Can Do-It-Yourself Continuous Glucose Monitoring Technology Improve Fear of Hypoglycaemia in Parents of Children Affected by Type 1 Diabetes? *Journal of Diabetes and Metabolic Disorders* **19(2)**, 1647-1658 (2020).

7 Evers, P. D. et al. Wearable Cardioverter-Defibrillators in Pediatric Cardiomyopathy: A Cost-Utility Analysis. *Heart Rhythm* **17(2)**, 287-293 (2020).

8 Gist, K. M. et al. Cosmetic Outcomes and Quality of Life in Children with Cardiac Implantable Electronic Devices. *PACE - Pacing and Clinical Electrophysiology* **42(1)**, 46-57 (2019).

9 Greenhill, D. A., Poppino, K. F., Jo, C. H. & Sucato, D. J. Intraoperative Prone Radiographs During Scheuermann Kyphosis Correction Closely Estimate Standing Thoracic and Lumbar Parameters at 2 Years. *Journal of Pediatric Orthopaedics* **40(10)**, 581-586 (2020).

10 Huhn, F., Jordening, M. G., Ernst, G. & Lange, K. Real World Use of Cgm among Adolescents and Young Adults with Type 1 Diabetes (T1d): Reduced Burden, but Little Interest in Data-Analyses. *Pediatric Diabetes* **20(Supplement 28)**, 191 (2019).

11 Kreutzer, J. et al. Comparison of the Investigational Device Exemption and Post-Approval Trials of the Melody Transcatheter Pulmonary Valve. *Catheterization and Cardiovascular Interventions* **98(2)**, E262-E274 (2021).

12 Lee, M., Holmes-Walker, J. & Farrell, K. The Impact of Continuous Glucose Monitoring in Youth with Type 1 Diabetes, Aged 16-21. *Diabetes Conference: 79th Scientific Sessions of the American Diabetes Association, ADA* **68** (2019).

13 Lertudomphonwanit, T., Jain, V. V., Sturm, P. F. & Patel, S. Periapical-Dropout Screws Strategy for 3-Dimensional Correction of Lenke 1 Adolescent Idiopathic Scoliosis in Patients Treated by Posterior Spinal Fusion. *Clinical Spine Surgery* **32(8)**, E359-E365 (2019).

14 Liu, W. et al. Three-Dimensional-Printed Intercalary Prosthesis for the Reconstruction of Large Bone Defect after Joint-Preserving Tumor Resection. *Journal of Surgical Oncology* **121(3)**, 570-577 (2020).

15 Maksimovic, M. et al. The Effect of Vertebral Body Tethering on Spine Range of Motion in Adolescent Idiopathic Scoliosis: A Pilot Study. *Spine Deformity* (2022).

16 Massa, G. G., Gys, I., Bevilacqua, E., Wijnands, A. & Zeevaert, R. Comparison of Flash Glucose Monitoring with Real Time Continuous Glucose Monitoring in Children and Adolescents with Type 1 Diabetes Treated with Continuous Subcutaneous Insulin Infusion. *Diabetes Research and Clinical Practice* **152**, 111-118 (2019).

17 Mathew, J. L., Kumar, R. & Harit, D. Continuous Vs. Intermittent Insulin Delivery in Children and Adolescents with Type 1 Diabetes Mellitus. *Indian Pediatrics* **56(7)**, 595-602 (2019).

18 McElhinney, D. B. et al. Endocarditis after Transcatheter Pulmonary Valve Replacement. *Journal of the American College of Cardiology* **72(22)**, 2717-2728 (2018).

19 Nimri, R. et al. Insulin Dose Optimization Using an Automated Artificial Intelligence-Based Decision Support System in Youths with Type 1 Diabetes. *Nat Med* **26**, 1380-1384 (2020).

20 Photopoulos, G. et al. Reliability of Radiographic Assessment of Growth Modulation from Anterior Vertebral Body Tethering Surgery in Pediatric Scoliosis. *Spine Deformity* (2022).

21 Piona, C. et al. High Glycemic Variability Is Associated with Worse Continuous Glucose Monitoring Metrics in Children and Adolescents with Type 1 Diabetes. *Pediatric Diabetes* **22(SUPPL 30)**, 98-99 (2021).

22 Piona, C. et al. Long-Term Glycemic Control and Glucose Variability Assessed with Continuous Glucose Monitoring in a Pediatric Population with Type 1 Diabetes: Determination of Optimal Sampling Duration. *Pediatric Diabetes* **21(8)**, 1485-1492 (2020).

23 Pook, C., Kuhn, E., Singh, A. & Kovach, J. Contact Force Ablation of Accessory Pathways in Pediatric Patients. *Journal of Cardiovascular Electrophysiology* **32(2)**, 370-375 (2021).

24 Sawyer, A., Sobczak, M., Forlenza, G. P. & Alonso, G. T. Glycemic Control in Relation to Technology Use in a Single-Center Cohort of Children with Type 1 Diabetes. *Diabetes Technology and Therapeutics* **24(6)**, 409-415 (2022).

25 Schierloh, U. et al. Effect of Predicted Low Suspend Pump Treatment on Improving Glycaemic Control and Quality of Sleep in Children with Type 1 Diabetes and Their Caregivers: The Quest Randomized Crossover Study. *Trials* **19(1) (no pagination)** (2018).

26 Sharma, A. et al. Clinico-Radiological and Functional Outcome of Difficult Talipes Equinovarus Deformity Corrected with an Ilizarov Fixator. *Journal of Foot and Ankle Surgery* **61(4)**, 719-725 (2022).

27 Sharma, V. et al. Pulmonary Valve Replacement: A Single-Institution Comparison of Surgical and Transcatheter Valves. *Annals of Thoracic Surgery* **106(3)**, 807-813 (2018).

28 Shin, J. et al. Study Design of Randomized, Adaptive Trial in Adult and Pediatric Patients with Type 1 Diabetes Using Hybrid Closed Loop Versus Control (Csii, Mdi or Sap). *Diabetes Technology and Therapeutics* **20(Supplement 1)**, A84 (2018).

29 Somali, M., Paschou, S. A. & Mouslech, Z. Insulin Pumps Use in Greece: Efficacy and Safety Data from 140 Patients with Type 1 Diabetes Mellitus. *Diabetes Research and Clinical Practice* **160 (no pagination)** (2020).

30 Szadkowska, A. et al. Impact of Factory-Calibrated Freestyle Libre System with New Glucose Algorithm Measurement Accuracy and Clinical Performance in Children with Type 1 Diabetes During Summer Camp. *Pediatric Diabetes* **22(2)**, 261-270 (2021).

31 Teoh, I. H. et al. Real-Time Continuous Glucose Monitoring Benefits Glycaemic Control in Adolescents and Young Adults with Type 1 Diabetes Irrespective of Insulin Delivery Modality. *Diabetic Medicine* **38(SUPPL 1)**, 1 (2021).

32 Verhofste, B. P. et al. Growth-Friendly Spine Surgery in Arthrogryposis Multiplex Congenita. *Journal of Bone and Joint Surgery* **103(8)**, 715-726 (2021).

33 Xu, L. et al. Is Growth-Friendly Surgical Treatment Superior to One-Stage Posterior Spinal Fusion in 9- to 11-Year-Old Children with Congenital Scoliosis? *Clinical Orthopaedics and Related Research* **478(10)**, 2375-2386 (2020).

34 Zhao, Z. et al. Plastic Lengthening Amputation with Vascularized Bone Grafts in Children with Bone Sarcoma: A Preliminary Report. *World Journal of Surgical Oncology* **18(1) (no pagination)** (2020).

35 Zhao, Z. et al. Outcomes of Fixed-Hinged Knee Prosthesis for Distal Femoral Osteosarcoma in Skeletally Immature Patients: First Results. *Journal of Knee Surgery* **(no pagination)** (2021).
36 Breton, M. D. et al. Continuous Glucose Monitoring and Insulin Informed Advisory System with Automated Titration and Dosing of Insulin Reduces Glucose Variability in Type 1 Diabetes Mellitus. *Diabetes Technology and Therapeutics* **20(8)**, 531-540 (2018).
37 Buyuk, A. F., Milbrandt, T. A., Mathew, S. E. & Larson, A. N. Measurable Thoracic Motion Remains at 1 Year Following Anterior Vertebral Body Tethering, with Sagittal Motion Greater Than Coronal Motion. *The Journal of bone and joint surgery* **American volume. 103(24)**, 2299-2305 (2021).
38 Canbolat, N., Basaran, I., Altun, D., Akgul, T. & Buget, M. I. Postoperative Pain in Adolescent Idiopathic Scoliosis Surgery: A Randomized Controlled Trial. *Pain Physician* **25(4)**, E589-E596 (2022).
39 DiMeglio, L. A. et al. Time Spent Outside of Target Glucose Range for Young Children with Type 1 Diabetes: A Continuous Glucose Monitor Study. *Diabetic Medicine* **37(8)**, 1308-1315 (2020).
40 Gimenez, M. et al. Revisiting the Relationships between Measures of Glycemic Control and Hypoglycemia in Continuous Glucose Monitoring Data Sets. *Diabetes Care* **41(2)**, 326-332 (2018).
41 Horke, A. et al. Early Results from a Prospective, Single-Arm European Trial on Decellularized Allografts for Aortic Valve Replacement: The Arise Study and Arise Registry Data. *Eur J Cardiothorac Surg* **58**, 1045-1053 (2020).
42 Hu, M. et al. Intraoperative Halo-Femoral Traction During Posterior Spinal Arthrodesis for Adolescent Idiopathic Scoliosis Curves between 70degree and 100degree: A Randomized Controlled Trial. *Journal of Neurosurgery: Spine* **36(1)**, 78-85 (2022).
43 Klonoff, D. C. et al. A Randomized, Multicentre Trial Evaluating the Efficacy and Safety of Fast-Acting Insulin Aspart in Continuous Subcutaneous Insulin Infusion in Adults with Type 1 Diabetes (Onset 5). *Diabetes, Obesity and Metabolism* **21(4)**, 961-967 (2019).
44 Koriem, E., El-Mahy, M. M., Atiyya, A. N. & Diab, R. A. Comparison between Supercharged Ulnar Nerve Repair by Anterior Interosseous Nerve Transfer and Isolated Ulnar Nerve Repair in Proximal Ulnar Nerve Injuries. *Journal of Hand Surgery* **45(2)**, 104-110 (2020).
45 Lawton, J. et al. Broadening the Debate About Post-Trial Access to Medical Interventions: A Qualitative Study of Participant Experiences at the End of a Trial Investigating a Medical Device to Support Type 1 Diabetes Self-Management. *AJOB Empir Bioeth* **10**, 100-112 (2019).
46 Moniotte, S., Owen, M., Barrea, T., Robert, A. & Lysy, P. A. Outcomes of Algorithm-Based Modifications of Insulinotherapy During Exercise in Mdi Vs Insulin Pump-Treated Children with Type 1 Diabetes: Results from the Tread-Diab Study. *Pediatric Diabetes* **18(8)**, 925-933 (2017).
47 Prahalad, P. et al. Teamwork, Targets, Technology, and Tight Control in Newly Diagnosed Type 1 Diabetes: The Pilot 4t Study. *Journal of Clinical Endocrinology and Metabolism* **107(4)**, 998-1008 (2022).
48 Schoelwer, M. J. et al. Predictors of Time-in-Range (70-180 Mg/Dl) Achieved Using a Closed-Loop Control System. *Diabetes Technol Ther* **23**, 475-481 (2021).
49 Sinisterra, M. et al. Patterns of Continuous Glucose Monitor Use in Young Children Throughout the First 18 Months Following Type 1 Diabetes Diagnosis. *Diabetes Technology and Therapeutics* **23(11)**, 777-781 (2021).
50 Tucholski, K., Sokolowska, M., Tucholska, D., Kaminska, H. & Jarosz-Chobot, P. Assessment of Optimal Insulin Administration Timing for Standard Carbohydrates-Rich Meals Using Continuous Glucose Monitoring in Children with Type 1 Diabetes: A Cross-over Randomized Study. *Journal of Diabetes Investigation* **10(5)**, 1237-1245 (2019).
51 Verbeeten, K. C. et al. Motivational Stage at Continuous Glucose Monitoring (Cgm) Initiation in Pediatric Type 1 Diabetes Is Associated with Current Glycemic Control but Does Not Predict Future Cgm Adherence or Glycemic Control. *Canadian Journal of Diabetes* **45(5)**, 466-472.e464 (2021).

52 Yang, D. et al. Effects of Metformin Added to Insulin in Adolescents with Type 1 Diabetes: An Exploratory Crossover Randomized Trial. *Journal of Diabetes Research* **2020 (no pagination)** (2020).

53 Burckhardt, M. A. et al. Use of Continuous Glucose Monitoring Trends to Facilitate Exercise in Children with Type 1 Diabetes. *Diabetes Technology and Therapeutics* **21(1)**, 51-55 (2019).

54 Drago, F. et al. Radiofrequency Catheter Ablation of Left-Sided Accessory Pathways in Children Using a New Fluoroscopy Integrated 3d-Mapping System. *Europace* **19(7)**, 1198-1203 (2017).

55 Etemadifar, M. R., Andalib, A., Rahimian, A. & Nodushan, S. M. H. T. Cobalt Chromium-Titanium Rods Versus Titanium-Titanium Rods for Treatment of Adolescent Idiopathic Scoliosis; Which Type of Rod Has Better Postoperative Outcomes? *Revista da Associacao Medica Brasileira* **64(12)**, 1085-1090 (2018).

56 Foltynski, P., Ladyzynski, P., Pankowska, E. & Mazurczak, K. Efficacy of Automatic Bolus Calculator with Automatic Speech Recognition in Patients with Type 1 Diabetes: A Randomized Cross-over Trial. *J Diabetes* **10**, 600-608 (2018).

57 Frielitz, F. S. et al. How Does Video-Counseling Work under Real-Life Conditions? Results from the Vidiki Study, a Multicenter, Controled Study Evaluating the Impact of Monthly Video Consultations for Children with Type 1 Diabetes Using a Continuous Glucose Monitoring System. *Pediatric Diabetes* **20(Supplement 28)**, 178 (2019).

58 Hilliard, M. E. et al. Type 1 Doing Well: Pilot Feasibility and Acceptability Study of a Strengths-Based Mhealth App for Parents of Adolescents with Type 1 Diabetes. *Diabetes Technology and Therapeutics* **22(11)**, 835-845 (2020).

59 Horn, J., Hvid, I., Huhnstock, S., Breen, A. B. & Steen, H. Limb Lengthening and Deformity Correction with Externally Controlled Motorized Intramedullary Nails: Evaluation of 50 Consecutive Lengthenings. *Acta Orthopaedica* **90(1)**, 81-87 (2019).

60 Karakostas, T., Swaroop, V. & Dias, L. Foot Pressure Analysis to Assess Severe Equinoplanovalgus Foot Deformity Correction Using the Calcaneal-Cuboid-Cuneiform Osteotomy. *Developmental Medicine and Child Neurology* **61(Supplement 3)**, 208 (2019).

61 Lastikka, M., Oksanen, H., Helenius, L., Pajulo, O. & Helenius, I. Comparison of Circular and Sagittal Reinforced Rod Options on Sagittal Balance Restoration in Adolescents Undergoing Pedicle Screw Instrumentation for Idiopathic Scoliosis. *World Neurosurgery* **127**, e1020-e1025 (2019).

62 Mazurczak, K., Pankowska, E., Ladyzynski, P. & Foltynski, P. The First Use of Bolus Calculator with Speech Analyzer. *Journal of Diabetes Science and Technology* **11(1)**, 7-11 (2017).

63 Paul, M. et al. Non-Contact Sensing of Neonatal Pulse Rate Using Camera-Based Imaging: A Clinical Feasibility Study. *Physiological measurement* **41(2)**, 024001 (2020).

64 Popkov, D., Dolganova, T., Mingazov, E., Dolganov, D. & Kobyzev, A. Combined Technique of Titanium Telescopic Rods and External Fixation in Osteogenesis Imperfecta Patients: First 12 Consecutive Cases. *Journal of Orthopaedics* **22**, 316-325 (2020).

65 Rajasekaran, S., Bhushan, M., Aiyer, S., Kanna, R. & Shetty, A. P. Accuracy of Pedicle Screw Insertion by Airo Intraoperative Ct in Complex Spinal Deformity Assessed by a New Classification Based on Technical Complexity of Screw Insertion. *European Spine Journal* **27(9)**, 2339-2347 (2018).

66 Reichert, J. C., Hofer, A., Matziolis, G. & Wassilew, G. I. Intraoperative Fluoroscopy Allows the Reliable Assessment of Deformity Correction During Periacetabular Osteotomy. *Journal of Clinical Medicine* **11(16) (no pagination)** (2022).

67 Schatz, D. et al. Insulin Dose Optimization Using an Automated Artificial Intelligence-Based Decision Support System in Youths with Type 1 Diabetes. *Nature Medicine* **26(9)**, 1380-1384 (2020).

68 Verheul, F. J. M. G. et al. Effectiveness of a Crossover Prosthetic Foot in Active Children with a Congenital Lower Limb Deficiency: An Explorative Study. *Prosthetics and Orthotics International* **44(5)**, 305-313 (2020).

69 Bakaniene, I., Urbonaviciene, G., Janaviciute, K. & Prasauskiene, A. Effects of the Ineventions Method on Gross Motor Function in Children with Spastic Cerebral Palsy. *Neurologia i Neurochirurgia Polska* **52(5)**, 581-586 (2018).
70 Hilmisson, H., Berman, S. & Magnusdottir, S. Sleep Apnea Diagnosis in Children Using Software-Generated Apnea-Hypopnea Index (Ahi) Derived from Data Recorded with a Single Photoplethysmogram Sensor (Ppg): Results from the Childhood Adenotonsillectomy Study (Chat) Based on Cardiopulmonary Coupling Analysis. *Sleep and Breathing* **24(4)**, 1739-1749 (2020).
71 Janson, C. M. et al. A Prospective Assessment of Optimal Mechanical Ventilation Parameters for Pediatric Catheter Ablation. *Pediatric Cardiology* **40(1)**, 126-132 (2019).
72 Malluccio, C. L. et al. Silver-Impregnated, Antibiotic-Impregnated or Non-Impregnated Ventriculoperitoneal Shunts to Prevent Shunt Infection: The Basics Three-Arm Rct. *Health Technology Assessment* **24(17)**, 1-114 (2020).
73 Mogenot, M. et al. Efficacy, Tolerability, and Safety of an Innovative Medical Device for Improving Oral Accessibility During Oral Examination in Special-Needs Patients: A Multicentric Clinical Trial. *PLoS ONE* **15(9 September) (no pagination)** (2020).
74 Murina, F. et al. Efficacy and Safety of a Novel Vaginal Medical Device in Recurrent Bacterial Vaginosis: A Multicenter Clinical Trial. *Minerva Ginecol* **72**, 292-298 (2020).
75 Sharif, H. et al. Feasibility Study of a New Magnetic Resonance Imaging Mini-Capsule Device to Measure Whole Gut Transit Time in Paediatric Constipation. *Journal of pediatric gastroenterology and nutrition* **71(5)**, 604-611 (2020).
76 Sharma, M. et al. Impact of a Novel Hypothermia Alert Device on Death of Low Birthweight Babies at Four Weeks: A Non-Randomized Controlled Community-Based Trial. *Indian Pediatrics* **57(4)**, 305-309 (2020).
77 Tayyib, N. et al. The Effectiveness of the Skincare Bundle in Preventing Medical-Device Related Pressure Injuries in Critical Care Units: A Clinical Trial. *Advances in skin & wound care* **34(2)**, 75-80 (2021).
78 Auquilla-Clavijo, P. E. et al. Comparative Study between Subcutaneous and Endovascular Defibrillator Recipients Regarding Tolerance to the Implant Procedure and Perception of Quality of Life. *Arquivos Brasileiros de Cardiologia* **116(6)**, 1139-1149 (2021).
79 Kuck, K. H. et al. Catheter Ablation Versus Best Medical Therapy in Patients with Persistent Atrial Fibrillation and Congestive Heart Failure: The Randomized Amica Trial. *Circ Arrhythm Electrophysiol* **12**, e007731 (2019).
80 Rohella, D. et al. Comparison of Fixed-Bearing Prosthesis Vs. Mobile-Bearing Prosthesis in Total Knee Arthroplasty among Indian Patients. *International Journal of Pharmaceutical and Clinical Research* **14(8)**, 7-12 (2022).
81 Turagam, M. K. et al. Automated Noncontact Ultrasound Imaging and Ablation System for the Treatment of Atrial Fibrillation: Outcomes of the First-in-Human Value Trial. *Circ Arrhythm Electrophysiol* **13**, e007917 (2020).
82 Blair, J. et al. Continuous Subcutaneous Insulin Infusion Versus Multiple Daily Injections in Children and Young People at Diagnosis of Type 1 Diabetes: The Scipi Rct. *Health Technology Assessment* **22(42)**, vii-111 (2018).
